# Selective Toxicity Mechanisms of Carbon Nanotubes and Near-Infrared Light Wave on the Colon and Hepatoma Cancer Cells

**DOI:** 10.5812/ijpr-157296

**Published:** 2024-12-23

**Authors:** Farshad Lotfollahzadeh, Nasim Nobari, Fatemeh Ghanbary, Hossein Hooshyar

**Affiliations:** 1 Department of Physic, Mahabad Branch, Islamic Azad University, Mahabad, Iran; 2 Department of Chemistry, Mahabad Branch, Islamic Azad University, Mahabad, Iran

**Keywords:** Stress Oxidative, Cell Death Signaling, Nanoparticle, Cytotoxicity

## Abstract

**Background:**

Cancer is a devastating disease with varying mortality rates and severe treatment side effects. Researchers are exploring alternative treatments that target cancer cells with high selectivity and minimal side effects. Photothermal therapy has shown promise as one such treatment option.

**Objectives:**

Single-walled carbon nanotubes (SWCNTs) and multi-walled carbon nanotubes (MWCNTs) can penetrate cellular membranes and convert near-infrared light into heat for photothermal therapy (PTT).

**Methods:**

In a recent study, carbon nanotubes (CNTs) were used in combination with PTT to treat HT29 and PCL/PRF/5 cancerous cells for different durations (6, 12, 24, 48, and 72 hours). The cytotoxicity of each treatment was evaluated through MTT assay, reactive oxygen species (ROS) analysis, lipid peroxidation, lysosomal membrane integrity, and protein carbonyl analysis.

**Results:**

The study found that SWCNTs, MWCNTs, and PTT each individually had a significant cytotoxic effect on cancer cells. However, when used together, they were even more effective in destroying cancer cells. Combining SWCNTs with PTT resulted in the highest level of cytotoxicity.

**Conclusions:**

These findings suggest that using CNTs, especially SWCNTs, in combination with PTT shows promise for treating cancer.

## 1. Background

The impact of cancer on human life is devastating, resulting in physical and emotional pain for both victims and their loved ones. Despite advances in medical technology and treatments, cancer remains a leading cause of death worldwide, with its mortality rate greatly influenced by various factors such as the type and stage of the disease, as well as the availability of healthcare and therapy services (1, 2).

Cancer treatment is a complex and multifaceted field, with many different approaches. While cancer treatment can be effective at destroying cancerous cells, it often has significant side effects and limitations. For example, chemotherapy, one of the most common forms of cancer treatment, uses powerful drugs to reduce cancer cells. However, this therapy induces a range of symptoms such as fatigue, nausea, hair loss, and an increased risk of infections due to its impact on healthy cells in the body (3, 4). Radiation therapy is another approach that uses powerful energy to target and damage cancer cells (5). However, limitations of radiation therapy include fatigue, skin irritation, and damage to surrounding tissues (6). Surgery to remove tumors or other cancerous growths is also common, but it can be risky due to bleeding, infection, and other complications (7). Furthermore, common cancer treatments may not be effective for certain types of cancer or for patients with specific genetic mutations, in addition to the side effects they cause. To overcome the limitations of traditional cancer treatment methods, innovative strategies are being explored.

Photothermal therapy (PTT) uses laser light to selectively heat and kill cancer cells. The method involves using a laser to target cancer cells, which emits near-infrared light absorbed by nanoparticles like gold or carbon nanotubes. These nanoparticles convert the light into heat, causing a rise in temperature in the cancer cells and ultimately leading to their destruction. One of the significant advantages of PTT is its high selectivity, meaning it targets cancer cells while sparing healthy ones. This makes PTT a promising alternative to cancer therapies like chemotherapy and radiation therapy, which often lack specificity and have systemic absorption (8, 9). Moreover, the combination of PTT with immunotherapy or chemotherapy increases effectiveness compared to when these therapies are used individually (10). Additionally, PTT is a non-invasive technique that doesn't require surgery, making it less traumatic for patients. Furthermore, PTT can be performed repeatedly without causing additional harm to the patient's body, and its effects can be monitored in real-time using imaging techniques. When further researched and developed, PTT has the potential to be a powerful tool in the fight against cancer, providing a safe and effective alternative to current treatments (11).

Nanoparticles (NPs) are an emerging area of cancer research that shows potential for improving the effectiveness and reducing the side effects of cancer treatment. These particles, which range in size from 1 to 100 nanometers, can be designed to target specific cancer cells or other structures within the body. Targeting specific cancer cells or other structures within the body can be achieved through the design of these particles, which can range in size from 1 to 100 nanometers (12, 13). Additionally, nanoparticles can be engineered to bypass biological barriers such as the blood-brain barrier, allowing them to reach tumors that might otherwise be difficult to treat. Studies suggest that nanoparticles hold great promise for improving outcomes for cancer patients (13, 14).

Carbon nanotubes (CNTs) are cylindrical structures made up of carbon atoms arranged in a unique pattern, forming a tube-like structure with remarkable mechanical, thermal, and electrical properties. These nanotubes have diameters on the order of nanometers and can be several millimeters long. Due to their high strength, low weight, and excellent conductivity, CNTs have become essential materials in various fields, from electronics to energy storage to biomedical engineering. Carbon nanotubes have shown immense promise as a potential tool for cancer therapy due to their unique properties. One of the most important abilities of CNTs is their ability to penetrate cellular membranes easily, allowing them to deliver therapeutic agents directly into cancer cells (15). Additionally, CNTs can absorb and convert near-infrared light into heat, which can be used for PTT (16).

## 2. Objectives

In this study, we applied single-wall and multi-wall carbon nanotubes (SWCNTs and MWCNTs) in combination with PTT to destroy colorectal and hepatocarcinoma cancer cells. For this purpose, we treated cancerous cells with single-walled carbon nanotubes (SWCNTs) and multi-walled carbon nanotubes (MWCNTs) and exposed the cells to an infrared laser for PTT. We evaluated the level of activation of the oxidative stress pathway by the nanotube structure and PTT, both individually and in combination, across different cancer cell lines.

## 3. Methods

### 3.1. Materials

In this study, we utilized SWCNTs and MWCNTs with a diameter of 80 nm and a concentration of 25 mg/mL (please add the concentration of the CNT solution that you used) (purchased from Sigma Aldrich), an 808 nm near-infrared (NIR) continuous wave (CW) laser (Connet Fiber Optics Co. Ltd), HT29 cell line (human colorectal adenocarcinoma cell line), PCL/PRF/5 cell line (hepatoma cell line), MTT (3-(4,5-dimethylthiazol-2-yl) -2,5-diphenyltetrazolium bromide), 10% fetal bovine serum (FBS), DMEM/F12 medium [Dulbecco's Modified Eagle Medium (DMEM) and Ham's Nutrient Mixture F-12 (F-12)], acridine orange solution, and penicillin/streptomycin (100 units/mL of penicillin and 100 μg/mL of streptomycin). Transmission electron microscopy (TEM).

### 3.2. Methodology

#### 3.2.1. Cell Culture

HT29 cell lines were cultured in DMEM/F12 medium (Gibco, USA) containing 10% FBS and penicillin/streptomycin (100 units/mL of penicillin and 100 μg/mL of streptomycin) and incubated at 37°C with 5% CO_2_. The cells were used after 2 - 6 passages. The cell culture protocol for the PCL/PRF/5 cell line is similar to that of the HT29 cell line (17). For evaluating cytotoxicity, 6 groups of cells were prepared by treating cells with SWCNTs, MWCNTs, and PTT, including control (without any treatment), TIR (treated with PTT), TS (treated with SWCNTs), TM (treated with MWCNTs), TIR-S (treated with PTT in combination with SWCNTs), and TIR-M (treated with PTT in combination with MWCNTs). IC_50_ concentrations were used for SWCNTs (approximately 125 mg/L) and MWCNTs (approximately 20 μg/mL).

#### 3.2.2. Photothermal Therapy of Cancer Cells

For PTT of cancer cells, 5 × 10^4^ HT29 and PCL/PRF/5 cells in 6 groups (control, TIR, TS, TM, TIR-S, and TIR-M) were prepared on a 12-well plate and incubated at 37°C in a humidified atmosphere containing 5% CO_2_. The cancer cells were exposed to an 808 nm NIR laser for 10 minutes with a power density of 1 W/cm².

#### 3.2.3. Cell Viability

To evaluate the viability of cancer cells, 5 × 10^4^ HT29 and PCL/PRF/5 cells in 6 groups (control, TIR, TS, TM, TIR-S, and TIR-M) were prepared on a 12-well plate and incubated at 37°C in a humidified atmosphere containing 5% CO_2_. After 6, 12, 24, 48, and 72 hours of treatment, based on a previous study, 50 μL of MTT solution was added to the plate. After 4 hours, 100 μL of DMSO was added to each well, and the plate was incubated for an additional 10 minutes. The absorbance was read at 570 nm using an ELISA reader (Infinte 200 M, Tecan, Basel, Switzerland) (18).

#### 3.2.4. Lysosomal Membrane Integrity Assay

To assess the stability of lysosomal membranes, we employed the acridine orange redistribution assay. Acridine orange is a fluorescent dye that can selectively stain acidic compartments within cells, including lysosomes. In a healthy cell, acridine orange accumulates within lysosomes and emits green fluorescence. However, when lysosomal damage occurs, acridine orange leaks into the cytoplasm, where it binds to other acidic organelles such as mitochondria, leading to a redistribution of the dye and an increase in red fluorescence (19). For this purpose, after 6, 12, 24, 48, and 72 hours of cellular treatment, we obtained a prestained cell suspension (control, TIR, TS, TM, TIR-S, and TIR-M) containing acridine orange (5 mM) from the incubation medium, centrifuged it for 1 minute at 800 g, and resuspended the cell pellet in DMEM. To remove any fluorescence dye that may have been present in the media, we performed two washes. Finally, we used a fluorimeter set (Fluorimetry, Shimadzu RF-5000, Japan; digital scale, Japan; Shaker, REAX2000, Iran) at λexcitation/λemission = 495/530 nm to measure the distribution of the fluorescent dye within the cell suspension (20).

#### 3.2.5. Reactive Oxygen Species Assay

In this experiment, isolated cells (1 × 10^6^ cells/mL) were suspended in respiration buffer. Afterwards, DCFH-DA (10 μM) was added to the wells of the plate and incubated for 15 minutes at 37°C. Subsequently, a fluorimeter set (Fluorimetry, Shimadzu RF-5000, Japan; digital scale, Japan; Shaker, REAX2000, Iran) was used to measure reactive oxygen species (ROS) levels at λexitation = 488 nm and λemission = 527 nm (20).

#### 3.2.6. Lipid Peroxidation Assay

The amount of thiobarbituric acid reactive substances (TBARS) formation was used to measure the content of lipid peroxidation (LPO) in all groups. A concentration of 1 × 10^6^ cells/mL was used for each group to measure LPO content. Finally, an ELISA reader (Infinite 200 M, Tecan, Basel, Switzerland) at 532 nm was used to measure the LPO content. The test was repeated three times for each sample (20).

#### 3.2.7. Protein Carbonyl Assay

First, the proteins were precipitated by adding an equal volume of 20% TCA and then centrifuged at 11,000 × g for 5 minutes. Next, cells (1 × 10^6^ cells/well) were resuspended in 2, 4-dinitrophenylhydrazine solution (10 mmol/L) at room temperature for 15 - 30 minutes. Afterward, 20% TCA was added, and the samples were centrifuged at 11,000 × g for 3 minutes. Finally, an ELISA reader (Infinite 200 M, Tecan, Basel, Switzerland) at 450 nm was used to measure the protein carbonyl content (20).

## 4. Results

### 4.1. Characterization of Carbon Nanotubes

**Figure 1.**
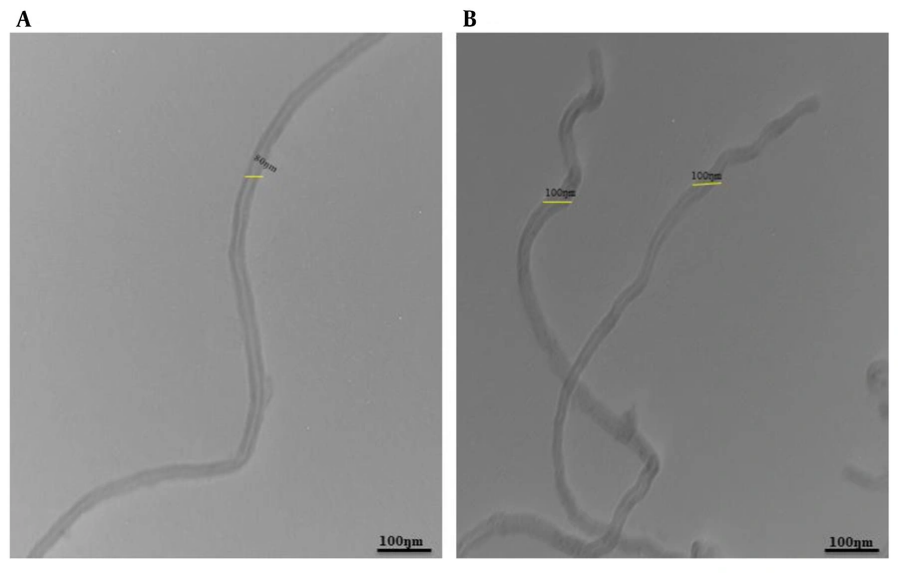
Transmission electron microscopy (TEM) micrograph of A, single-wall carbon nanotubes; and B, multi-wall carbon nanotubes (CNTs)

For the characterization of CNTs, we utilized TEM. Figure 1A and B show the TEM micrographs of SWCNTs and MWCNTs, respectively.

### 4.2. Cell Viability

**Figure 2.**
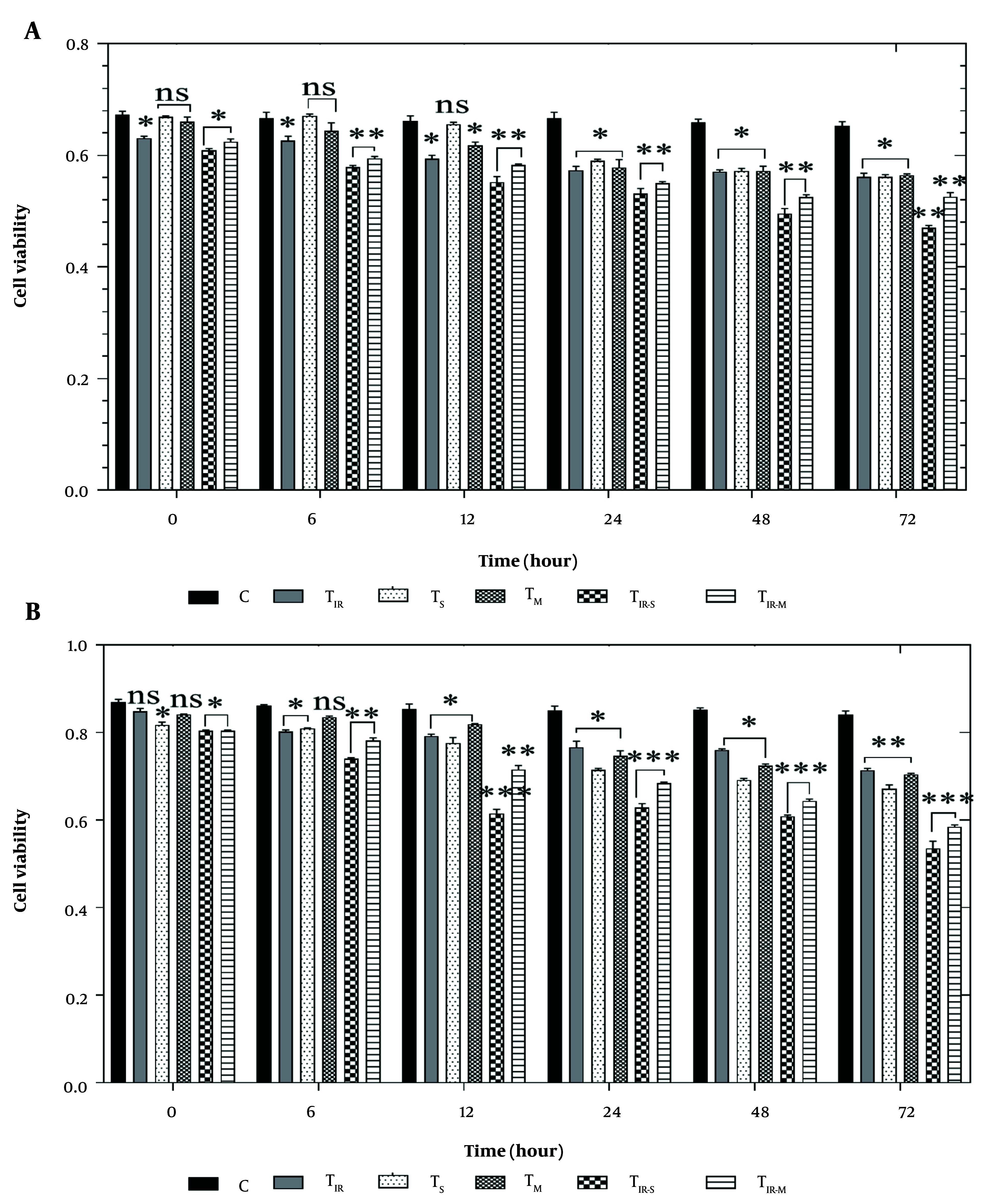
A, MTT assay results of H29; and B, PCL/PRF/5 cancer cells in 6 groups including control, TIR, TS, TM, TIR-S, and TIR-M. The cells were treated with photothermal therapy (PTT) and carbon nanotubes (CNTs) for different durations of 0, 6, 12, 24, 48 and 72 h. Data shown as mean ± SD. *, ** and *** represent P < 0.05, P < 0.01 and P < 0.001 (respectively) vs control group. ns: No significant.

To evaluate the effect of PTT and CNT treatment on the HT29 and PCL/PRF/5 cancer cells, the MTT assay was applied to six groups of cells, including control, TIR, TS, TM, TIR-S, and TIR-M. The results of the MTT assay on the HT29 cell line in these six groups, after 6, 12, 24, 48, and 72 hours of PTT and CNT treatment, are shown in Figure 2A. Cell viability was significantly reduced in TIR, TIR-S, and TIR-M (P < 0.05) after the start time, and in TIR (P < 0.05), TIR-S (P < 0.01), and TIR-M (P < 0.01) after 6 and 12 hours. After 48 and 72 hours, cell viability was reduced in TIR, TS, and TM (P < 0.05), and in TIR-S and TIR-M (P < 0.01) compared to the control group (Figure 2A). 

Additionally, the cell viability results for PCL/PRF/5 cells in these six groups, after 6, 12, 24, 48, and 72 hours of treatment, are shown in Figure 2B. Cell viability was significantly reduced in TS, TIR-S, and TIR-M (P < 0.05) after the start time, and in TIR, TS (P < 0.05), TIR-S, and TIR-M (P < 0.01) after 6 hours. After 12 hours, cell viability was reduced in TIR, TS, and TM (P < 0.05), and in TIR-S and TIR-M (P < 0.001). After 24, 48, and 72 hours, cell viability was reduced in TIR, TS, TM (P < 0.05), and in TIR-S and TIR-M (P < 0.001) compared to the control group (Figure 2B). 

### 4.3. Lysosomal Membrane Integrity Assay

**Figure 3.**
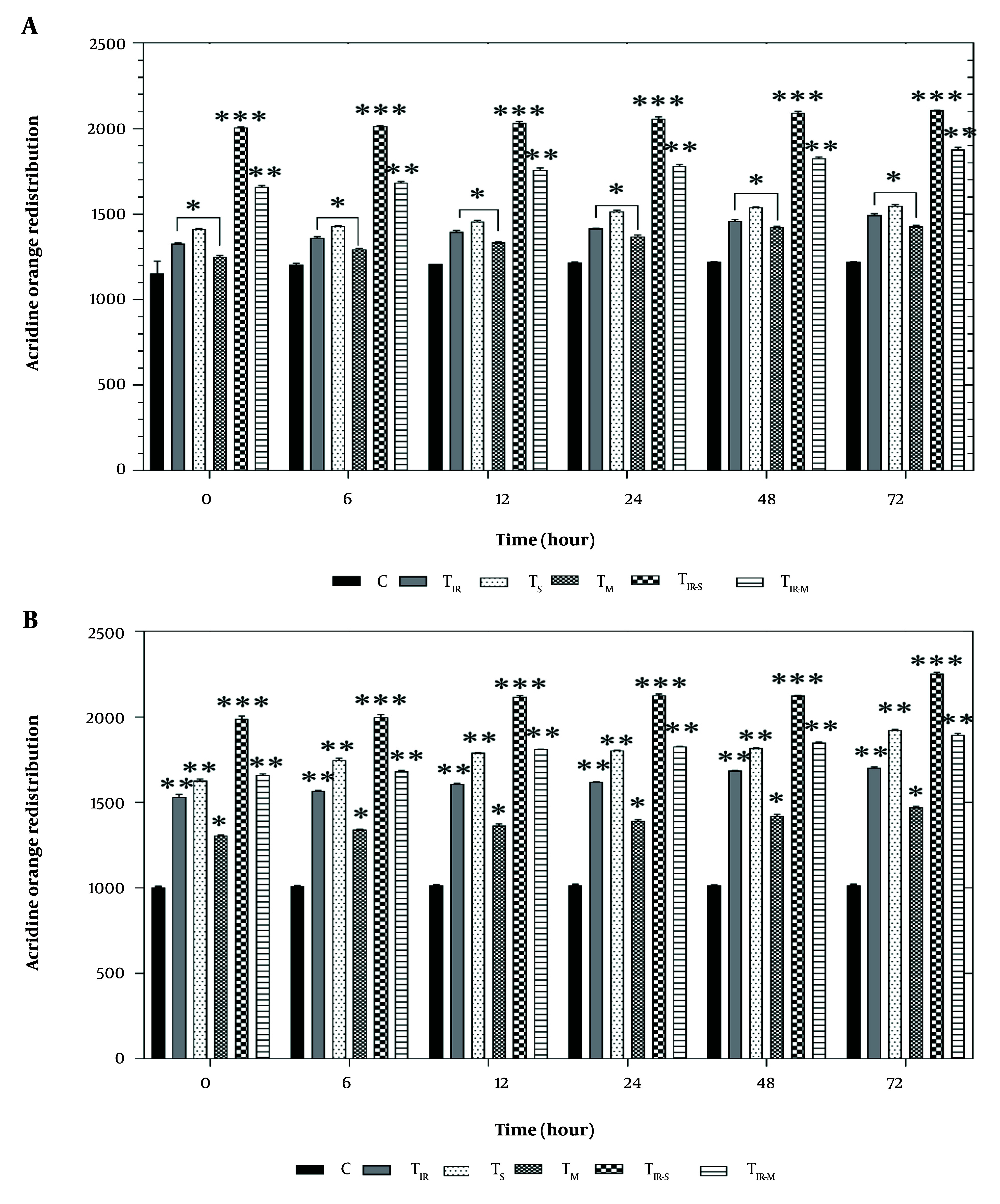
Lysosomal membrane integrity evaluation via A, acridine orange redistribution results of H29; and B, PCL/PRF/5 cancer cells in 6 groups including control, TIR, TS, TM, TIR-S, and TIR-M. The cells were treated with PTT and carbon nanotubes (CNTs) for different durations of 0, 6, 12, 24, 48 and 72 h. Data shown as mean ± SD. *, ** and *** represent P < 0.05, P < 0.01and P < 0.001 (respectively) vs control group.

Figure 3A shows the results of the acridine orange redistribution assay on the HT29 cell line in six groups after 6, 12, 24, 48, and 72 hours of PTT and CNT treatment. Lysosomal membrane damage was significantly observed in TIR, TS, and TM (P < 0.05), TIR-S (P < 0.001), and TIR-M (P < 0.01) after 6, 12, 24, 48, and 72 hours compared to the control group. 

Additionally, the results of the acridine orange redistribution assay on PCL/PRF/5 cells in the six groups after 6, 12, 24, 48, and 72 hours of treatment are shown in Figure 3B. Lysosomal damage was significantly increased in TIR, TS (P < 0.01), TM (P < 0.05), TIR-S (P < 0.001), and TIR-M (P < 0.01) after 6, 12, 24, 48, and 72 hours compared to the control group.

### 3.4. Reactive Oxygen Species Assay

**Figure 4.**
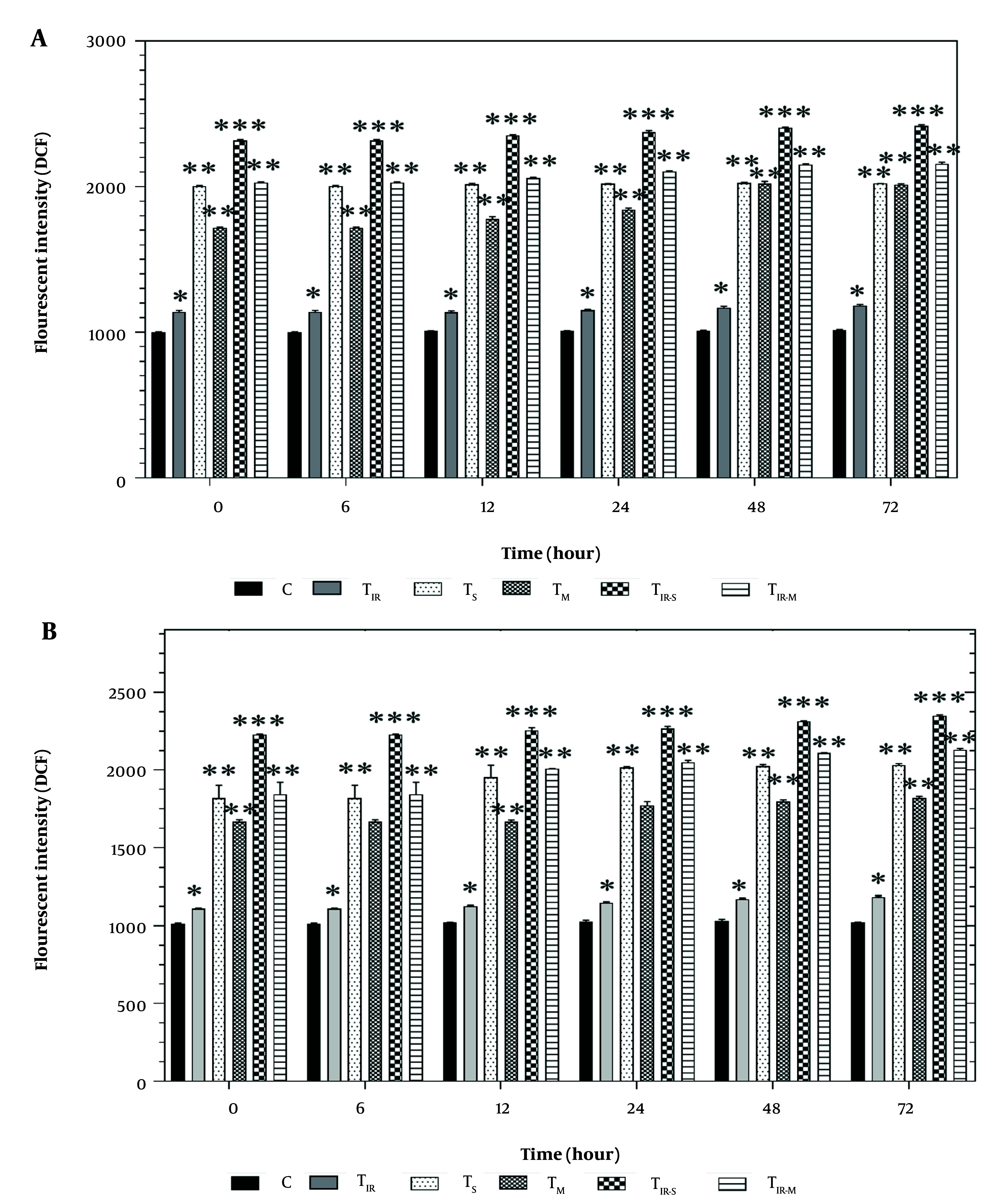
Reactive oxygen species (ROS) evaluation via A, DCFH-DA results of H29; and B, PCL/PRF/5 cancer cells in 6 groups including control, TIR, TS, TM, TIR-S, and TIR-M. The cells were treated with photothermal therapy (PTT) and carbon nanotubes (CNTs) for different durations of 0, 6, 12, 24, 48 and 72 h. Data shown as mean ± SD. *, ** and *** represent P < 0.05, P < 0.01and P < 0.001 (respectively) vs control group.

Figure 4A shows the results of the DCFH-DA assay on the HT29 cell line in six groups after 6, 12, 24, 48, and 72 hours of PTT and CNT treatment. Reactive oxygen species levels were significantly increased in TIR (P < 0.05), TS, TM, TIR-M (P < 0.01), and TIR-S (P < 0.001) after 6, 12, 24, 48, and 72 hours compared to the control group.

Additionally, the results of the ROS assay on PCL/PRF/5 cells in the six groups after 6, 12, 24, 48, and 72 hours of treatment are shown in Figure 4B. Reactive oxygen species levels were significantly increased in TIR (P < 0.05), TS, TM, TIR-M (P < 0.01), and TIR-S (P < 0.001) after 6, 12, 24, 48, and 72 hours compared to the control group.

### 4.5. Lipid Peroxidation Assay

**Figure 5.**
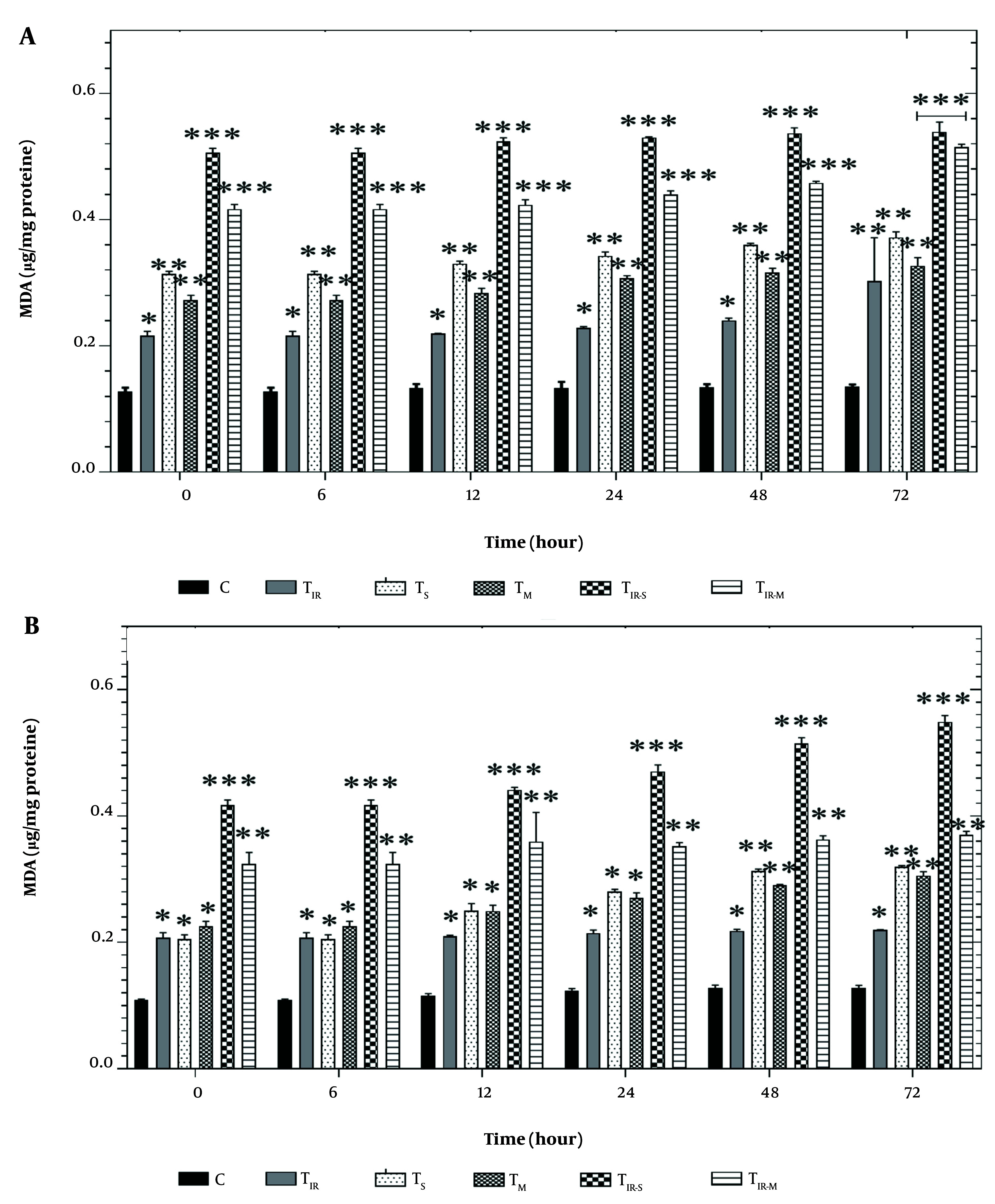
Lipid peroxidation (LPO) assay by A, TBARS results of H29; and B, PCL/PRF/5 cancer cells in 6 groups including control, TIR, TS, TM, TIR-S, and TIR-M. The cells were treated with photothermal therapy (PTT) and carbon nanotubes (CNTs) for different durations of 0, 6, 12, 24, 48 and 72 h. Data shown as mean ± SD. *, ** and *** represent P < 0.05, P < 0.01and P < 0.001 (respectively) vs control group.

Figure 5A shows the results of the LPO assay on the HT29 cell line in six groups after 6, 12, 24, 48, and 72 hours of PTT and CNT treatment. Lipid peroxidation levels were significantly induced in TIR (P < 0.05), TS, TM (P < 0.01), TIR-M, and TIR-S (P < 0.001) after 6, 12, 24, 48, and 72 hours compared to the control group.

Additionally, the results of the LPO assay in PCL/PRF/5 cells are shown in Figure 5B. Lipid peroxidation levels were significantly altered in TIR, TS, TM (P < 0.05), TIR-M (P < 0.001), and TIR-S (P < 0.01) groups after 6, 12, 24, 48, and 72 hours compared to the control group.

### 4.6. Protein Carbonyl Assay

**Figure 6.**
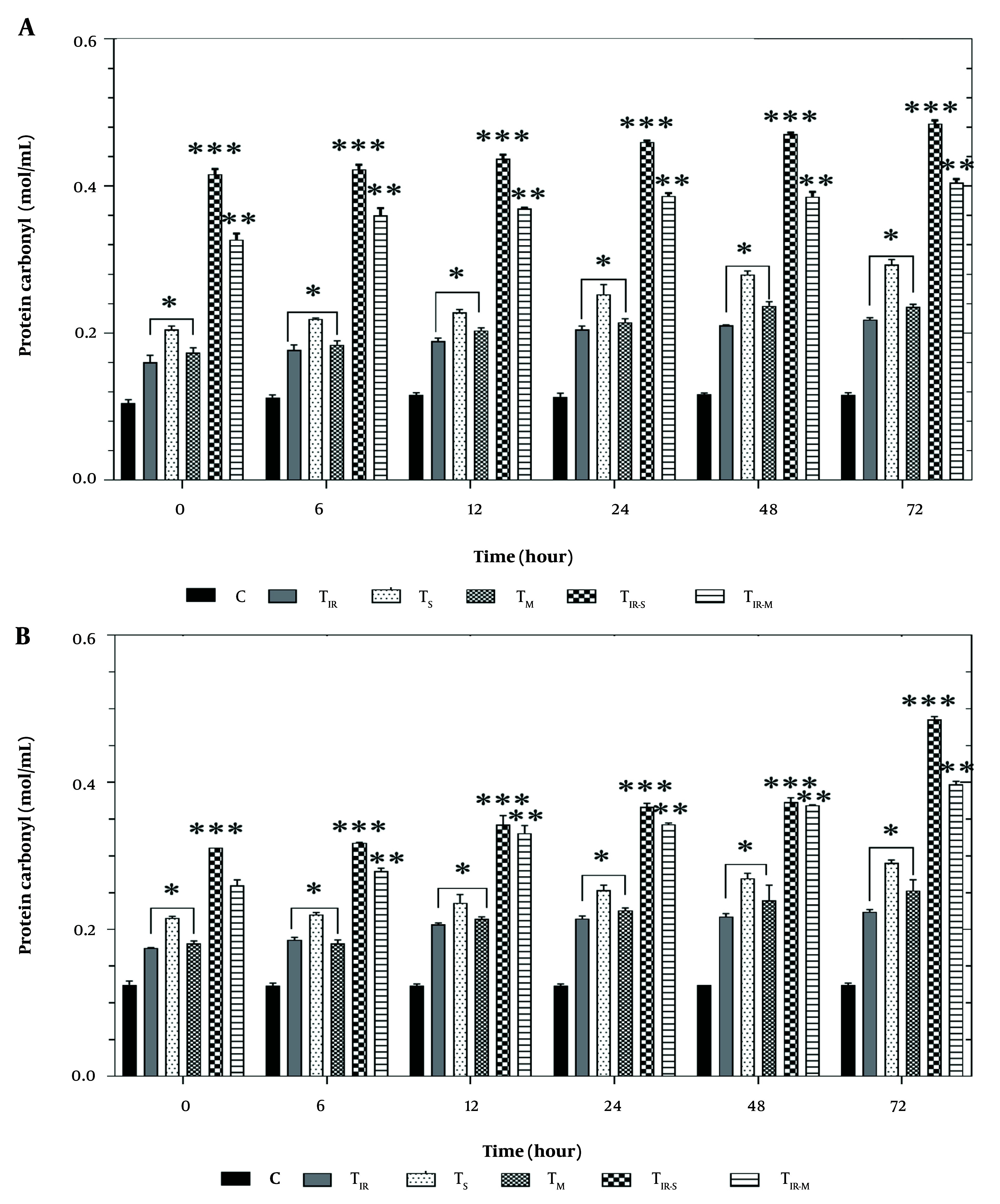
A, Protein carbonyl results of H29; and B, PCL/PRF/5 cancer cells in 6 groups including control, TIR, TS, TM, TIR-S, and TIR-M. The cells were treated with photothermal therapy (PTT) and carbon nanotubes (CNTs) for different durations of 0, 6, 12, 24, 48 and 72 h. Data shown as mean ± SD. *, ** and *** represent P < 0.05, P < 0.01and P < 0.001 (respectively) vs control group.

Figure 6A shows the results of the protein carbonyl assay on the HT29 cell line in six groups after 6, 12, 24, 48, and 72 hours of PTT and CNT treatment. Protein carbonyl levels were significantly increased in TIR, TS, and TM (P < 0.05), TIR-M (P < 0.001), and TIR-S (P < 0.01) after 6, 12, 24, 48, and 72 hours compared to the control group.

Similarly, the results of the protein carbonyl assay on PCL/PRF/5 cells are shown in Figure 6B. Protein carbonyl levels were significantly increased in TIR, TS, and TM (P < 0.05), TIR-M (P < 0.001), and TIR-S (P < 0.01) after 6, 12, 24, 48, and 72 hours compared to the control group.

## 5. Discussion

In this study, we examined the capability of using a mixture of SWCNTs and MWCNTs with PTT to destroy colorectal and hepatocarcinoma cancer cells. To achieve this, we treated the cancerous cells with both SWCNTs and MWCNTs and exposed them to an infrared laser for PTT. We assessed the cytotoxicity of the CNTs and PTT, both separately and in combination, with HT29 and PCL/PRF/5 cancer cells. 

The MTT test was used to assess the impact of PTT and CNT treatment on HT29 and PCL/PRF/5 cancer cells, with a total of six cell groups being tested (control, TIR, TS, TM, TIR-S, and TIR-M). Figure 2A and B present the outcomes of the MTT assay on HT29 and PCL/PRF/5 cell lines after treatment with PTT and CNTs for 6, 12, 24, 48, and 72 hours. The results showed that the viability of HT29 and PCL/PRF/5 cells decreased over time (Figure 2A and B). After 6 hours of treatment, a decrease in cell viability was observed only in three groups of HT29 cells (TIR, TIR-S, and TIR-M) and in two groups of PCL/PRF/5 cells (TIR-S and TIR-M). Gradually, with increasing time, cell viability decreased, and after 72 hours of treatment, cell viability reached its minimum level. Consult with the outcomes of the MTT assay on HT29 and PCL/PRF/5 cell lines after the treatment of the cells with PTT and CNTs for 6, 12, 24, 48, and 72 hours. The results showed that the viability of HT29 and PCL/PRF/5 cells decreased with increasing time (see Figures 2A and B). After 6 hours of treatment, a decrease in cell viability was observed only in 3 groups of HT29 cells, including TIR, TIR-S, and TIR-M, and in 2 groups of PCL/PRF/5 cells, including TIR-S and TIR-M. Gradually, with increasing time, cell viability decreased, and 72 hours after treatment, cell viability reached its minimum level.

Upon analysis, it was observed that the use of SWCNTs, MWCNTs, and PTT individually induced a significant decrease in cellular viability after 24 hours in both HT29 and PCL/PRF/5 cancer cells. However, when CNTs were used in combination with PTT for the treatment of cells, the toxicity levels were highest for the cells. This was evident from the figures, where the viability of the cells was the lowest in the TIR-S and TIR-M groups after 72 hours. It is worth noting that the TIR-S group of cells had lower cell viability than the TIR-M group at all examined time points, indicating that the toxicity of SWCNTs in combination with PTT was higher than all other groups significantly.

Some studies have confirmed the cytotoxicity of CNTs and PTT on cells. For example, Wang et al. investigated the cytotoxic effects of SWCNTs on PC12 cells, a type of neural cell line. The study found that SWCNTs caused significant cytotoxicity in PC12 cells, with the extent of cytotoxicity depending on the treatment time and concentration of the SWCNTs. They suggested that the toxicity was likely due to the induction of ROS (21, 22). Reddy et al. reported the effects of MWCNTs on HEK293 cells. Their study showed that MWCNTs led to cytotoxicity and oxidative stress in HEK293 cells, possibly through the generation of ROS. The researchers observed a dose-dependent increase in ROS production and a decrease in cell viability following exposure to MWCNTs (23).

They confirmed that cellular death induced through ROS, and the activation of the oxidative stress pathway caused modifications in mitochondrial characteristics and DNA damage (23). Li et al. evaluated the use of PTT to induce immunogenic cell death (ICD) in breast cancer cells using natural melanin nanoparticles (24). Li et al. found that PTT was effective in inducing ICD, resulting in improved anti-tumor immune responses and suppression of tumor growth. The use of natural melanin nanoparticles as the photothermal agent also provided a biocompatible and low-toxicity treatment option (24). This was evident from Figure 4A and B, where the ROS levels of the cells were altered for the TIR-S and TIR-M groups after all the time points. The TIR-S group had higher ROS than the TIR-M group at all tested times, indicating that the toxicity of SWCNTs in combination with PTT was higher than all other groups in the cancer cell line. 

It can be concluded that the production of ROS and lipid membrane damage contributed to the increase in MDA levels, while the oxidative damage to proteins caused by ROS in the cells contributed to the increase in protein carbonyl levels. Within the oxidative stress pathway, protein carbonyl is considered one of the critical markers and often indicates a decrease in protein function (24). There was significant protein peroxidation observed between the test groups and control cells at all times in the cancer cell line, and it was found that the TIR-S group had higher levels compared to the control group and TIR-M group at all times, indicating that the toxicity of SWCNTs in combination with PTT was higher than all other groups (Figure 6A and B).

Some studies have shown that PTT in combination with nanostructures is a promising method for cancer cell destruction. Jeyamohan et al. used a multifunctional SWCNT and MWCNT-based system for targeted drug delivery and PTT to kill cancer cells. The authors reported that this approach was highly effective in killing cancer cells in vitro, with minimal damage to healthy cells and side effects. The researchers suggest that this system could be a promising approach to cancer treatment, as it combines two powerful methods of cancer cell destruction (25). In another study, SWCNTs have been explored as a potential platform for targeted cancer therapy. In this approach, SWCNTs are functionalized with molecules that selectively bind to cancer cells, allowing for their accumulation within tumors. Once localized, the SWCNTs can be activated with light energy to generate heat, which damages cancer cells via photothermal therapy. Recently, researchers have developed mitochondria-targeting SWCNTs, which specifically accumulate in the mitochondria of cancer cells, where they cause increased damage and cell death (26). Additionally, some studies have shown that SWCNTs cause more apoptosis than MWCNTs (27, 28). These findings confirm the results of our study, demonstrating a decrease in cell viability of cancer cells using CNTs in combination with PTT, and the higher cytotoxicity of SWCNTs compared to MWCNTs in combination with PTT.

Figure 3A and B show the results of the acridine orange redistribution assay, which was conducted to evaluate lysosomal membrane integrity in 6 groups of HT29 and PCL/PRF/5 cells (control, TIR, TS, TM, TIR-S, and TIR-M) after 6, 12, 24, 48, and 72 hours of treatment. As shown in Figure 3A and B, after treatment with SWCNT, MWCNT, and PTT, acridine orange leaked into the cytoplasm, leading to the redistribution of the dye and an increase in red fluorescence over time. The highest amount of acridine orange redistribution was observed after 72 hours in all cell-treated groups (TIR, TS, TM, TIR-S, and TIR-M). This increase indicates a rise in lysosomal membrane damage and cytotoxicity caused by the treatment agents. 

Free radicals (such as O_2_− and H_2_O_2_) are produced through the normal function of the mitochondrial respiratory chain (20). Reactive oxygen species are involved in various physiological processes in mammalian cells. These active metabolites are produced in response to external stimuli through the activation of enzymes that generate pro-oxidants (21). We present the results of the ROS assay to evaluate the release of H_2_O_2_ in cells, which was carried out on 6 groups of HT29 and PCL/PRF/5 cells (control, TIR, TS, TM, TIR-S, and TIR-M) after 6, 12, 24, 48, and 72 hours of treatment (Figure 4A and B). As shown in Figure 4A and B, in both HT29 and PCL/PRF/5 cancer cells, after treatment with SWCNT, MWCNT, and PTT, the intensity of fluorescence increased over time. 

Reactive oxygen species are involved in damage to the lipid membrane. One of the consequences of lipid membrane damage is the disruption of the mitochondrial electron transfer chain, leading to the induction of cell death signaling (21). There appears to be a direct correlation between TBARS formation and LPO in cancer cell lines exposed to SWCNT, MWCNT, and PTT. As observed in Figure 5A and B, there is a time-dependent relationship between LPO and the treatment with SWCNT, MWCNT, and PTT in the cells. An increase in the level of TBARS formation can be associated with the release of pro-apoptotic proteins, such as cytochrome c, which is essential in initiating cell death signaling (21). Reactive oxygen species production and oxidative stress play an important role in damage to macromolecules (DNA, lipids, and proteins) in cells. Oxidative stress can disrupt pathways involved in metabolism, physiology, and pathology in cells. Additionally, ROS can lead to the production of free carbonyl proteins by altering the side chain of amino acids (24). Aging, stress in the endoplasmic reticulum and lysosome, and depletion of antioxidant capacity are consequences of protein carbonylation in tissues (21).

There was significant protein peroxidation between the control cells and the test group in cancer cell lines. Figure 6A and B show the results of the protein carbonyl assay on 6 groups of HT29 and PCL/PRF/5 cells (control, TIR, TS, TM, TIR-S, and TIR-M) after 6, 12, 24, 48, and 72 hours of treatment. The protein carbonyl level increased in both HT29 and PCL/PRF/5 cancer cells after treatment with SWCNT, MWCNT, and PTT. 

These results also confirm that the cytotoxicity of the combined treatment (TIR-S and TIR-M) on cancer cells is greater than that of the individual treatment agents (TIR, TS, TM). Similar to the MTT test results, the lysosomal membrane integrity assay results showed that the treatment of cells with SWCNTs in combination with PTT (TIR-S) caused the most cytotoxicity to both HT29 and PCL/PRF/5 cells compared to the other cell groups. 

Yang et al. evaluated the cytotoxicity of carbon nanohorns, a type of carbon nanotube, using the acridine orange redistribution assessment (29). Lysosomal activity is essential for maintaining cellular homeostasis, and lysosomal dysfunction has been implicated in various disease conditions, including lysosomal storage diseases (LSDs), neurodegeneration, autoimmune diseases, and cancer. Features of lysosomal dysfunction include changes in the expression and/or activity of lysosomal enzymes, changes in lysosomal size/number/pH/cellular positioning/motility, and changes in lysosomal membrane properties. The results showed that carbon nanohorns accumulate in lysosomes and cause lysosomal membrane permeabilization, leading to the release of cathepsins. This results in mitochondrial dysfunction and the production of ROS (21), ultimately causing apoptosis. Lysosomal dysfunction has been overlooked as an early cause of carbon nanotube toxicity, highlighting the need to consider lysosomal membrane permeabilization (LMP) in toxicity studies (29).

### 5.1. Conclusions

This research was designed to explore the potential of using SWCNTs and MWCNTs alongside PTT to eradicate colorectal and hepatocarcinoma cancer cells. To achieve this goal, we administered SWCNTs and MWCNTs to HT29 and PCL/PRF/5 cancer cells and exposed them to infrared laser treatment for PTT. We conducted both individual and combined assessments of the cytotoxicity of CNTs and PTT on HT29 and PCL/PRF/5 cancer cells. After treating six groups of cells, including control, TIR, TS, TM, TIR-S, and TIR-M, for 0, 6, 12, 24, 48, and 72 hours, the cell viability, lysosomal membrane integrity, reactive oxygen species, lipid peroxidation, and protein carbonyl assessments were analyzed, respectively. The results demonstrated that the utilization of SWCNTs, MWCNTs, and PTT individually had noteworthy cytotoxicity on HT29 and PCL/PRF/5 cancer cells, with the effect increasing over time. However, the application of combined treatment (SWCNTs and MWCNTs in combination with PTT) showed greater efficacy in the destruction of cancer cells. It should be noted that applying SWCNTs in combination with PTT caused the most cytotoxicity in cancerous cells compared to the other treatment groups. Based on the results obtained from our study, it can be concluded that combination therapy using CNTs, especially SWCNTs, with PTT can be a promising approach for cancer treatment. However, in vivo investigations are recommended for further exploration of this combination therapy.

## Data Availability

The dataset presented in the study is available on request from the corresponding author during submission or after publication.
